# Specific biomarkers and neurons distribution of different brain regions in largemouth bass (*Micropterus salmoides*)

**DOI:** 10.3389/fendo.2024.1385575

**Published:** 2024-04-30

**Authors:** Meijia Li, Leshan Yang, Lei Zhang, Qian Zhang, Ying Liu

**Affiliations:** ^1^ College of Biosystems Engineering and Food Science (BEFS), Zhejiang University, Hangzhou, China; ^2^ College of Fisheries and Life Science, Dalian Ocean University, Dalian, China; ^3^ Key Laboratory of Environment Controlled Aquaculture, Ministry of Education, Dalian, China; ^4^ College of Marine Technology and Environment, Dalian Ocean University, Dalian, China

**Keywords:** Micropterus salmoides, brain regions, morphology, transcriptome profiling, marker genes, neurons distribution

## Abstract

The brain regulates multiple physiological processes in fish. Despite this, knowledge about the basic structure and function of distinct brain regions in non-model fish species remains limited due to their diversity and the scarcity of common biomarkers. In the present study, four major brain parts, the telencephalon, diencephalon, mesencephalon and rhombencephalon, were isolated in largemouth bass, *Micropterus salmoides*. Within these parts, nine brain regions and 74 nuclei were further identified through morphological and cytoarchitectonic analysis. Transcriptome analysis revealed a total of 7153 region-highly expressed genes and 176 region-specifically expressed genes. Genes related to growth, reproduction, emotion, learning, and memory were significantly overexpressed in the olfactory bulb and telencephalon (OBT). Feeding and stress-related genes were in the hypothalamus (Hy). Visual system-related genes were predominantly enriched in the optic tectum (OT), while vision and hearing-related genes were widely expressed in the cerebellum (Ce) region. Sensory input and motor output-related genes were in the medulla oblongata (Mo). Osmoregulation, stress response, sleep/wake cycles, and reproduction-related genes were highly expressed in the remaining brain (RB). Three candidate marker genes were further identified for each brain regions, such as neuropeptide FF (*npff*) for OBT, pro-melanin-concentrating hormone (*pmch*) for Hy, vesicular inhibitory amino acid transporter (*viaat*) for OT, excitatory amino acid transporter 1 (*eaat1*) for Ce, peripherin (*prph*) for Mo, and isotocin neurophysin (*itnp*) for RB. Additionally, the distribution of seven neurotransmitter-type neurons and five types of non-neuronal cells across different brain regions were analyzed by examining the expression of their marker genes. Notably, marker genes for glutamatergic and GABAergic neurons showed the highest expression levels across all brain regions. Similarly, the marker gene for radial astrocytes exhibited high expression compared to other markers, while those for microglia were the least expressed. Overall, our results provide a comprehensive overview of the structural and functional characteristics of distinct brain regions in the largemouth bass, which offers a valuable resource for understanding the role of central nervous system in regulating physiological processes in teleost.

## Introduction

The brain is not only one of the most important but also the most complex organs in the body (1). Dysfunction of the brain can result in various diseases in humans, such as Parkinson’s disease, Alzheimer’s disease, depressive disorders, and so on (2, 3). Identifying the structural characteristics and functional roles of each brain region has been a research hotspot in the 21^st^ century. The fish brain also plays a central role in multiple physiological processes, such as controlling growth, development, reproduction, behavior, and stress response/adaptation, especially under artificial breeding environment (4). Compared with the rapid development of human brain science, knowledge of the structure and function of fish brain is still limited. A growing number of studies are exploring specific brain regions or nuclei, as well as the molecular mechanisms within the brains of various teleosts. However, due to the small size of the fish brain, coupled with the scarcity of biomarkers and the absence of advanced micromanipulation techniques, many studies continue to focus on the whole brain rather than targeting specific brain regions as their study object. This approach fails to accurately capture the specific functions of different brain regions (5). Therefore, there is an urgent need to further explore the microstructures and gene expression characteristics of different brain regions in fish.

Contrary to the brains of mammal, fish has evolved a distinct brain structure. The fish brain lacks a layered pallium and comprises four distinct regions: telencephalon, diencephalon, mesencephalon, and rhombencephalon (6). Presently, the microscopic anatomy has also been reported in some teleosts, such as zebrafish (*Danio rerio*), grass carp (*Ctenopharyngodon idellus*), sea bass (*Dicentrarchus labrax*), mudskipper (*Boleophthalmus pectinirostris*), tilapia (*Oreochromis mossambicus*), and African turquoise killifish (*Nothobranchius furzeri*) (7–12). Due to the rich diversity of teleost species and their habitats, their brains exhibit a remarkable array of morphological variations. For instance, there have been reported notable morphological differences in the brains of fish residing in seawater and freshwater environments, especially in the areas such as the olfactory bulb, optic tectum, and rhombencephalon (13). Similarly, deep-sea sharks, confronted with the scarcity of light, have evolved a highly advanced nonvisual sensor system, particularly the electrosensory system. This is accompanied by a notable hypertrophy of cerebellar-like structures in the medulla, which are brain areas associated with electrosensory function (14). There are significant differences in the positioning of nuclei within the internal structure among different fish species. For example, a fiber tract linking the granular layer of the valvula cerebelli and the torus longitudinalis was identified in sea bream (*Sparus aurata*), whereas it was absent in sea bass (*Dicentrarchus labrax*) (15).

Except for the distinction in fish brain microstructure, the detailed physiological functions of each brain region also need to be defined in fish. At present, most studies of brain structure and function in fish mainly focus on two brain regions: the telencephalon and the hypothalamus. The telencephalon has been characterized as the functional homologue of the mammalian limbic system, and is mainly implicated in memory and learning (6, 16). The hypothalamus is the starting point of endocrine axis, such as hypothalamic-pituitary-interrenal (HPI) and hypothalamic-pituitary-gonadal (HPG) axes, and plays important roles in regulating growth, reproduction, and stress response (17, 18). In contrast, the roles of the remaining brain regions of fish in regulating physiological functions are still poorly explored. As a model organism for studying complex brain disorders, zebrafish has been studied more deeply, and functions of its brain sub-regions are beginning to become clearer (19). Recently, cell types and gene expression characteristics of four brain regions (forebrain, optic tectum, hindbrain and the region underneath the optic tectum) have been revealed in zebrafish using single-cell transcriptome sequencing (20). However, compared with the study of zebrafish, the research progress on the function of different brain regions in farmed fish is still in its infancy.

Largemouth bass (*Micropterus salmoides*), a member of the teleost family, is a freshwater species popular with consumers worldwide (21). With increasing demand, the farming of largemouth bass is rapidly growing, and more attention is being paid on its biological processes, which directly correlate with economic benefit, such as growth, development, reproduction, immunity and stress response. All those biological processes are controlled by brain functions. However, the research on the anatomy, cytoarchitecture and function of different brain regions in largemouth bass is far from meeting the demand. In the present study, the cytoarchitecture and gene expression characterization of different brain regions in largemouth bass were explored with the objectives of (1) identifying the external and internal morphological structure of the largemouth bass brain, (2) investigating the gene expression pattern and identifying candidate markers for different brain regions, and (3) predicting the distribution patterns of neurotransmitter-type neurons and non-neuronal cells in different brain regions by analyzing the expression of their marker genes.

## Materials and methods

### Experimental animals and sampling

Adult largemouth basses *M*. *salmoides* (Youlu No. 3, accession number: GS-01-001-2018) with a body weight of approximately 450-500 g were obtained from Key Laboratory of Environment Controlled Aquaculture, Ministry of Education in Dalian Ocean University, Liaoning province, China. They were maintained in tanks (length of 4.5 m, width of 3.4 m, and height of 1.5 m) filled with filtered and aerated freshwater (temperature 18.0°C, pH 7.7, and dissolved oxygen ≥ 6.0 mg L^-1^), and fed twice daily with commercial feed (Tong Wei, China). Six largemouth bass were euthanized using an overdose concentration of 500 mg L^-1^ of tricaine methanesulfonate (MS-222) treatment as previous reports (22, 23). Of these, the brains of three fish were used to observe their external morphology and measure the dimensions (length, width, and depth) of different brain regions using a vernier caliper (accurate to 0.01 mm) according to the previous report (24). The whole brains of the remaining three fish were removed and fixed for slices preparation. For the RNA sequencing experiment, nine fish were randomly divided into three parallel groups and anesthetized. Six brain regions, including olfactory bulbs and telencephalon (OBT), hypothalamus (Hy), optic tectum (OT), cerebellum (Ce), medulla oblongata (Mo) and remaining brain (RB, consisting of the region wrapped in OT, excluding the valvula cerebelli) were collected, dissociated in TRIzol reagent and prepared for RNA extraction.

### Brain tissue slices preparation

Brain tissues were carefully separated from largemouth basses under an anatomical lens, fixed in Bouin’s fluid (G-CLONE, China) at room temperature for 24 h, then rinsed in 70% ethanol until nearly colorless, and subsequently dehydrated in 80%, 95%, 100% ethanol and xylene. The samples were then embedded in paraffin wax, serially sectioned (6 μm) using a rotary microtome (Leica, Germany), and mounted onto slides accordingly (25). After dewaxing and rehydrating, the slides were stained with hematoxylin-eosin (HE) and then scanned using a Pannoramic DESK slide scanner (3D HISTECH, Hungary). The location, size, shape, density, and staining intensity in different brain regions were used as major criteria to identify different nuclei. Symmetrical and representative sections were chosen for documentation and photographed. The boundaries of cell masses and fiber tracts were drawn on photographs, copied onto transparent paper, and digitized. The nomenclature used in this study is primarily based on that of previous studies (8, 9, 15, 26).

### RNA extraction, library preparation and RNA sequencing

Total RNA was extracted from collected samples using TRIzol (Invitrogen) according to the manufacturer’s protocol. Briefly, brain tissues of largemouth bass (50 mg) were homogenized in TRIzol reagent, and then chloroform and isopropanol were added to the tissue homogenate to separate the total RNA. Subsequently, mRNA was purified from the total RNA using poly-T oligo-attached magnetic beads, fragmented using divalent cations under elevated temperature and prepared for cDNA synthesis. The first and second strands cDNA were synthesized using a random hexamer primer, M-MuLV Reverse Transcriptase, and DNA Polymerase I with RNase H, respectively. After end repair and adaptor ligation, cDNA fragments of 370-420 bp in length were selected using the AMPure XP system (Beckman Coulter, Beverly, USA). PCR was then performed with Phusion High-Fidelity DNA polymerase, and the PCR products were purified for library construction. The library quality was assessed using the Agilent Bioanalyzer 2100 system and sequenced on an Illumina Novaseq platform (27).

### Differentially expressed genes and KEGG enrichment

After sequencing, adapter sequences and low-quality reads with *q*-value < 30 in raw sequencing data were filtered out, and clean data with high quality were obtained through the use of fastp software. These data were then mapped to the largemouth bass (*Micropterus salmoides*) reference genome (GCF_014851395.1_ASM1485139v1_genomic) using Hisat2 v2.0.5 with default parameters. The read counts for each gene were determined using feature Counts v1.5.0-p3 and the fragments per kilobase per million (FPKM) value for each gene was calculated (27). Based on the negative binomial distribution model, differentially expressed genes (DEGs) were analyzed using the edgeR package from the R Bioconductor suite. The *p*-value served as the threshold for the differential gene expression test. The threshold of the *p*-value in multiple tests was determined by the false discovery rate (FDR) value (28, 29). Kyoto encyclopedia of genes and genomes (KEGG) pathway enrichment analysis was implemented using the cluster Profiler R package, with correction for gene length bias. KEGG pathways with a *p-*value *<* 0.05 were considered significantly enriched (30). Principal component analysis (PCA) and heatmap were performed via the free online platform, Majorbio I-Sanger Cloud Platform (http://www.i-sanger.com).

### Highly and specifically expressed genes screened from each brain region

The screening of highly expressed genes and specifically expressed genes in each brain regions was conducted according to previous report (29). In the first step, region-highly expressed genes were identified by calculating differences in gene expression between the brain region of interest and the remaining samples. Only transcripts with an adjusted *p*-value < 0.05 were considered for further analysis. In the second step, genes for which the 25th percentile of expression in the brain region of interest was higher than the 90th percentile of expression in all other brain regions were deemed as region-highly expressed genes. Additionally, region-specifically expressed genes were further screened using those brain region’s highly expressed genes, with 10-fold higher FPKM values than those in other brain regions. The genes with the highest fold value were regarded as marker genes for each brain region.

### Predicting the distribution of neurons and non-neuronal cells in each brain region

The distributions of neurons and non-neuronal cells in different brain regions were predicted based on the expression levels of related marker genes. The marker genes for various types of neurons and non-neuronal cells were identified using data from model species zebrafish (20). Seven types of neurons were distinguished by specific marker genes: glutamatergic neurons by *slc17a6*, GABAergic neurons by *gad*, glycinergic neurons by *slc6a5*, dopaminergic neurons by *th*, serotonergic neurons by *tph2*, noradrenergic neurons by *dbh*, and cholinergic neurons by *chata*. These marker genes were screened from the brain transcriptomes of largemouth bass, and their expression levels in six brain regions were documented. Additionally, potential receptors for these seven neurotransmitters were screened based on the genome annotation of largemouth bass, and total expressions of each type receptor in six brain regions were also calculated and analyzed. The marker genes for five types of non-neuronal cells, including *olig2*, *sox10* (oligodendrocytes), *apoeb*, *mpeg1* (microglia), *rbp4* (endothelial cells), *cx43* (radial astrocytes), and *pcna* (neuroprogenitors) (20), were also identified in the transcriptomes of the largemouth bass brain. By comparing the expression levels of these marker genes across the six brain regions, predictions were made regarding the distribution of neurons and non-neuronal cells in different brain regions.

### Quantitative real-time PCR validation

Quantitative real-time PCR (qPCR) was conducted to validate the RNA-seq data, and the specific primers for 15 genes were designed using Primer 5 Premier 5.0. The sequences of these primers are listed in **Table 1**. The RNA samples used for qPCR amplifications were identical to those employed in the construction of the RNA-Seq library mentioned above. The qPCR was performed on the LightCycler^®^96 Real-Time PCR System (Roche, Basel, Switzerland) with SYBR Green I Master. The program of qPCR was as follow: 95°C for 30 s, 45 cycles of 95°C for 5 s and 60°C for 30 s. Dissociation curve analysis of the amplification products was performed at the end of each PCR reaction (consisting of 1 cycle of 95°C for 15 s, 60°C for 30 s, and 95°C for 15 s) to confirm that only one PCR product was amplified and detected. The relative expression level of each gene in each brain region was calculated according to the 2*^-^* ^ΔΔCt^ method and normalized to the endogenous control genes of β-actin (25).

**Table 1:** The sequence information of primers for qRCR.

Gene name	Forward primer (5’-3’)	Reverse primer (5’-3’)
*bhlhe23*	GGAGTCCAGAAAGAGAGGCT	GGGCGTAGGGGATAACAG
*npff*	TCGGACCTCAGAGAGAAACC	CCTCCCAATCACGAGAATGT
*pro-mch*	ACGAGGTGGCAGAAAACAGC	CAGGATGGGGATGGTCAGG
*nkx2*	CTGATCAAGATACTCCACAAG	GTAGACCTGAGCCTGCGAA
*viaat*	TGGACTCACATCGCAGCC	GCAGTGGGTAAGATAACAGC
*pax7*	TTGGCAAAAAAGATGACGAGG	GGTGAAGGTGGTGCGACTG
*aldoca*	ATGATTAGGGACAGGGGTAT	GCCAGTTTAGATGGATTGCT
*eaat1*	GCCATTACTCGGCAAAACCC	CCACAGGCAGCACGAAACG
*prph*	GCAGATGCGTGAAATGGAA	TTGAGGATGGGAACGGTAAT
*slc6a5*	CCTCTACTTTATCACACCGA	CTTCTCTATTGGCACTTTCA
*itnp*	AACTACCTGCTCACCCCTTG	GCTGGTTTGGTCATCTCCTT
*vtnp*	AGAACTACCTGCTCACCCCC	AGCTCCTTGTAGAGCCGTCA
*slc17a6a*	CAGCAGACAGGTGAGGTGAT	CTTGCCGTTTTGATGGATTG
*gad2*	CTAACATCCACTGCCAACACC	AGGGAACATCTTGAAGCGGG
*cx43*	ATGCTGGTGGTCTCACTGGTC	TTTAGGGGTGGGGCTCAAGG
*β-actin*	AAAGGGAAATCGTGCGTGAC	AAGGAAGGCTGGAAGAGGG

### Statistical analysis

All data were analyzed using statistical package for social sciences (SPSS) 16.0, and graphed using Origin 8.1. Statistical differences between two groups were assessed using Student’s t-test. One-way analysis of variance (ANOVA), followed by Duncan’s test, was used to compare differences among multiple groups. The differences were considered statistically significant at *p* < 0.05 and extremely significant at *p* < 0.01.

## Results

### Histological structure of different brain regions (parts-regions-nuclei)

**Figure 1:**
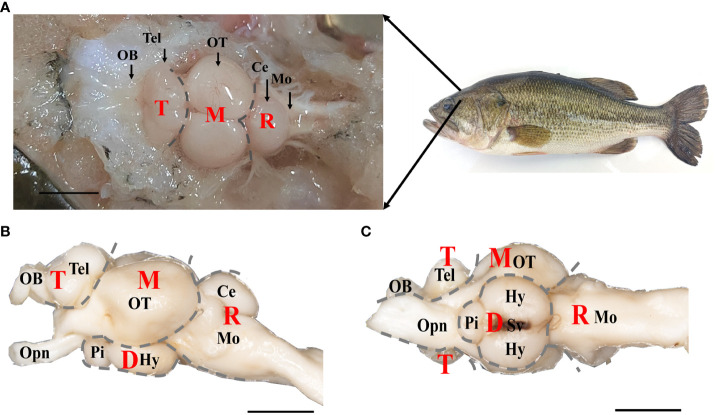
The gross anatomy of brain of largemouth bass. **(A)** Dorsal, **(B)** lateral, and **(C)** ventral views under anatomical lens were shown. T, Telencephalon. D, Diencephalon. M, Mesencephalon. R, Rhombencephalon. OB, Olfactory Bulbs. Tel, Telencephalon. OT, Optic Tectum. Ce, Cerebellum. Mo, Medulla Oblongata. Opn, Optic Nerve. Pi, Pituitary. Hy, Hypothalamus. Sv, Saccus Vasculosus. Scale bar = 5 mm in A, B, **(C)** Four brain parts (T, D, M and R) were labeled with gray dotted line. Notes, parts of structure in diencephalon brain region wrapped in OT were not labeled.

The brain tissues were dissected from euthanized fish, and the dorsal, lateral and ventral views of the brain were shown in **Figure 1**. It was composed of four parts: telencephalon (T), mesencephalon (M), diencephalon (D), and rhombencephalon (R), from rostral to caudal, which could be further divided into nine regions. The most rostral part of the brain was occupied by the olfactory bulb (OB) coupled with telencephalic hemispheres (Tel), which exhibited two parts symmetrically (**Figures 1A, B**). The mesencephalon was located caudal to the telencephalic hemispheres, mostly occupied by the optic tectum (OT) from the dorsal view (**Figure 1A**), and was connected to the eyes via the optic nerve (Opn) (**Figures 1B, C**). The diencephalon was located below the mesencephalon, including the hypothalamus (Hy) and pituitary (Pi), and connected to the saccus vasculosus (Sv) (**Figures 1B, C**). The rhombencephalon was connected to the mesencephalon and diencephalon in the caudal brain, and included cerebellum (Ce) and medulla oblongata (Mo) (**Figures 1A, B**). Refer to the previous study (24), the length, width, and depth of the different brain regions was measured. As shown in **Figure S1**, both the length and width of the OT region were highest among the brain regions of the olfactory bulb (OB), telencephalon (Tel), medulla oblongata (Mo), and corpus cerebelli (CCe) (*p* < 0.01). Furthermore, there was no significant difference in the depth of the OT region compared to the regions of Tel, Mo, and CCe (*p* > 0.01). These findings suggest that the size of OT region in largemouth bass is proportionally larger than other brain regions (**Figure S1**).

**Figure 2:**
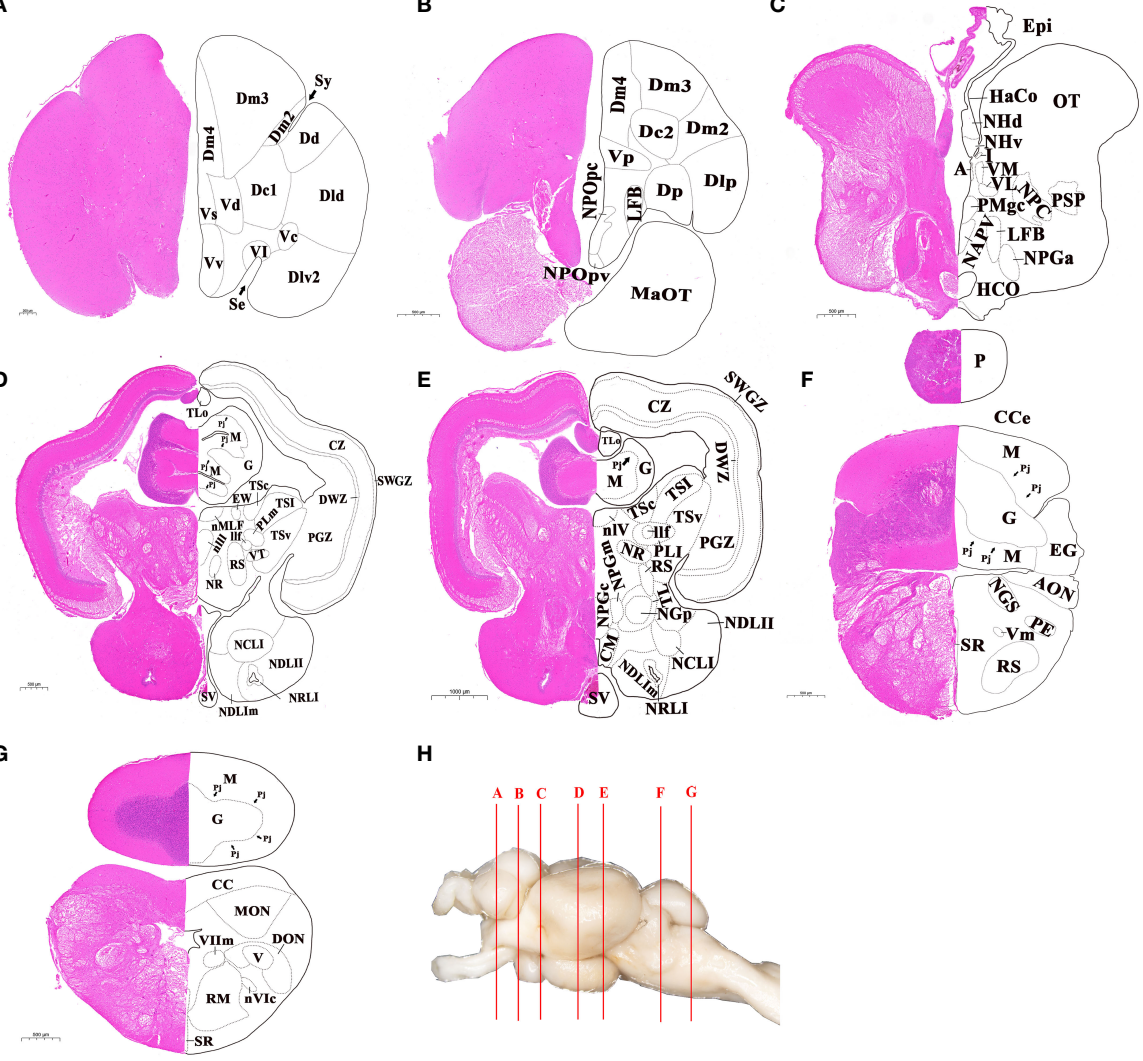
Cytoarchitecture of telencephalon, diencephalon, mesencephalon and rhombencephalon in largemouth bass. **(A, B)** Telencephalon. **(C-E)** Diencephalon. **(C-E)**, Mesencephalon. **(F, G)** Rhombencephalon. H, Lettered lines indicated the sites of the transverse sections in whole brain of largemouth bass. The abbreviations of nuclei identified in different brain regions were listed in ABBREVIATIONS. Scale bar = 200-1000 μm in **(A-G)**.

The whole brain was sectioned and transverse sections of brain were shown in **Figure 2**. According to the histological terminology of brain reported in previous studies (8, 9, 15, 26), a total of 14 subregions and 74 nuclei were identified in different brain regions. In the telencephalon, the brain regions of dorsal telencephalon and ventral telencephalon were subdivided, and there was a total of 16 nuclei identified. The dorsal telencephalon was subdivided into five nuclei: medial (Dm), dorsal (Dd), lateral (Dl), central (Dc), and posterior (Dp). The ventral telencephalon was subdivided into six nuclei: dorsal (Vd), ventral (Vv), supracommissural (Vs), postcommissural (Vp), lateral (Vl), and central (Vc) (**Figures 2A, B**). The diencephalon was subdivided into eight subregions, including the preoptic area, ventral thalamus, dorsal thalamus, epithalamus, hypothalamus, posterior tuberculum, synencephalon and pretectum, and 25 nuclei were included in it (**Figures 2C–E**). In the mesencephalon, two main subregions, the dorsal tectum and ventral tegmentum, were further divided, and 14 distinct nuclei were characterized. The dorsal tectum was subdivided into the optic tectum (OT) and torus longitudinalis (TLo), while the ventral tegmentum was subdivided into medial, central and lateral zones (**Figures 2D, E**). The rhombencephalon was subdivided into two main subregions: cerebellum and medulla oblongata, where 19 nuclei were further identified (**Figures 2F, G**). The cerebellum subregion was subdivided into three structures: valvula cerebelli, corpus cerebelli, and vestibulolateral lobe, where valvula cerebelli was present in the mesencephalic ventricle. The medulla oblongata was subdivided into five structures: reticular formation, area octavolateralis, somatomotor nuclei, visceromotor nuclei, and other rhombencephalic nuclei.

### Transcriptome characteristics for the six brain regions

**Figure 3:**
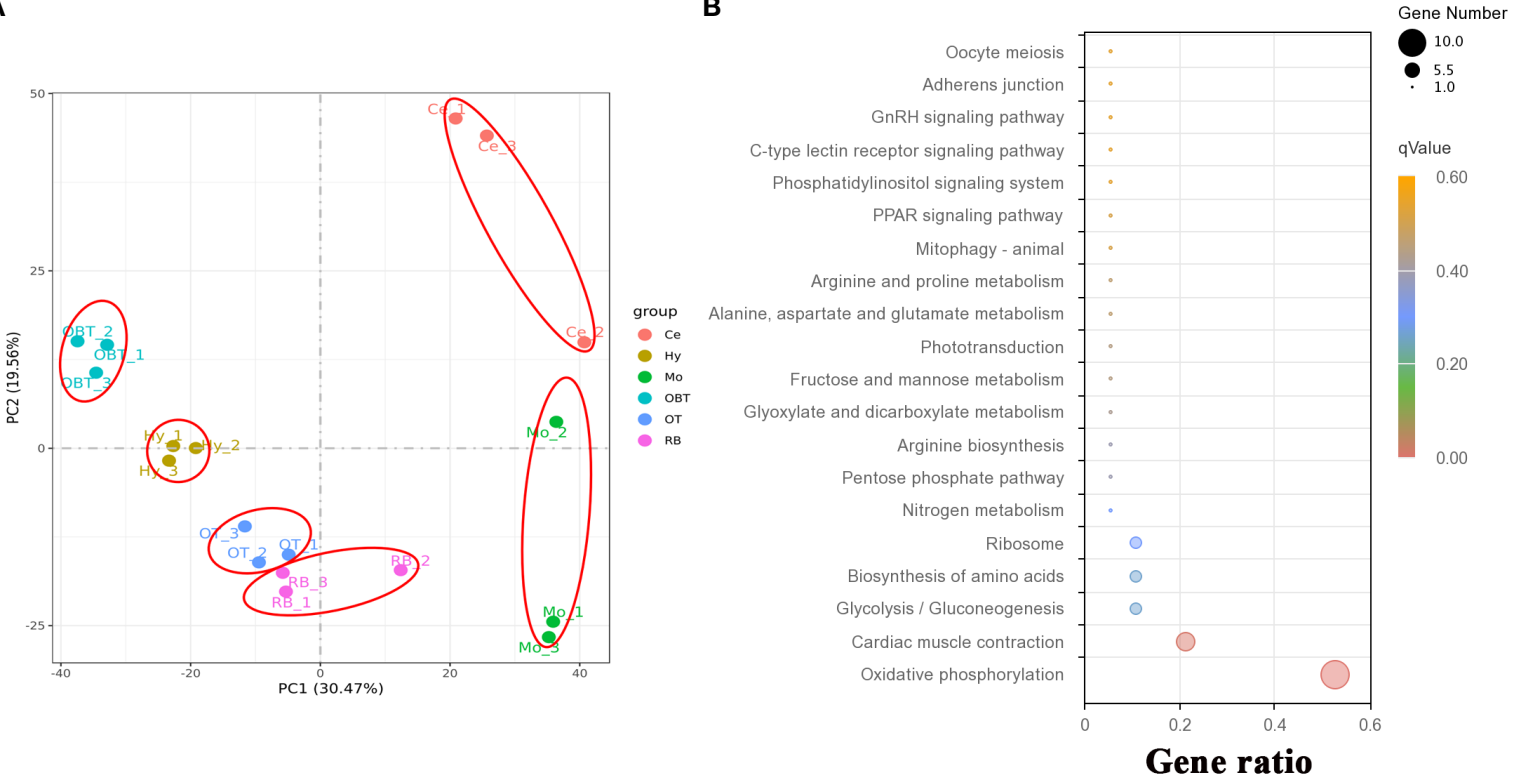
The correlation of transcriptome data and function prediction. **(A)** Results for the first two principal components (PCs) according to principal component analysis (PCA) using expressed genes from six brain regions. **(B)** Top 20 enriched KEGG pathways based on top 30 highly expressed genes in whole brain. OBT, telencephalon (including olfactory bulbs). Hy, Hypothalamus. OT, Optic Tectum. Ce, Cerebellum. Mo, Medulla Oblongata. RB, Remaining Brain. The gene ratio represented the ratio between the number of genes in a particular pathway and the number of all genes in this enrichment term. The sizes of the dots on these plots denoted the number of genes, while colors correspond to the *q* value range.

To investigate the gene expression patterns across different brain regions, transcriptional fragment libraries were constructed using samples from six distinct brain regions: the telencephalon (including olfactory bulbs, OBT), hypothalamus (Hy), optic tectum (OT), cerebellum (Ce), medulla oblongata (Mo), and the remaining brain (RB). Specifically, the RB region, which comprises the region wrapped in the OT, is located in the caudal of telencephalon, ventral of mesencephalon, dorsal of hypothalamus, and rostral of medulla oblongata. As depicted in the middle of **Figures 2C–E**, the RB region not only encompasses the subregion of the ventral tegmentum in the mesencephalon, but also incorporates the subregions such as the preoptic area, ventral thalamus, dorsal thalamus, posterior tuberculum, synencephalon, and pretectum in the diencephalon. A total of 808.97 million raw reads were generated, and after rigorous filtering to eliminate low-quality data, a comparable number of clean reads were obtained for each brain region. These clean reads were then aligned to the genome of the largemouth bass. The unique mapping rates for the18 libraries ranged from 88.98% to 89.97%, indicating a high level of sequence alignment and data reliability (**Table 2**). To assess the similarity between the three biological replicates, principal component analysis (PCA) was performed. The contribution ratio of the first principal component (PC1) and second principal component (PC2) was 30.47% and 19.56%, respectively. The PCA analysis revealed that the three biological replicates from the same brain region clustered together, indicating strong reproducibility within each group. Moreover, the six distinct brain regions exhibited significant differences, highlighting distinct transcriptional profiles among different brain regions (**Figure 3A**).

**Table 2:** Summary of transcriptome sequencing data.

Sample	Raw reads (10^6^)	Clean reads (10^6^)	Mapping reads (10^6^)	Mapping rate (%)	Unique mapping reads (10^6^)	Unique mapping rate (%)
**OBT_1**	41.36	40.65	38.51	94.74	36.54	89.89
**OBT_2**	46.33	45.57	43.07	94.53	40.81	89.55
**OBT_3**	44.56	43.74	41.28	94.39	39.04	89.26
**Hy_1**	42.40	41.70	39.34	94.34	37.39	89.66
**Hy_2**	41.84	41.20	38.88	94.36	36.98	89.75
**Hy_3**	44.47	43.78	41.15	93.98	39.04	89.16
**OT_1**	43.66	43.01	40.84	94.95	38.70	89.97
**OT_2**	44.54	43.80	41.43	94.59	39.14	89.35
**OT_3**	46.11	45.32	42.84	94.55	40.55	89.48
**Ce_1**	44.51	43.78	41.42	94.62	39.08	89.27
**Ce_2**	44.62	43.93	41.67	94.85	39.49	89.88
**Ce_3**	44.33	43.70	41.32	94.57	39.01	89.28
**Mo_1**	45.58	44.78	42.45	94.79	40.24	89.86
**Mo_2**	47.08	46.33	43.99	94.94	41.62	89.84
**Mo_3**	45.84	45.20	42.79	94.67	40.48	89.56
**RB_1**	45.49	44.74	42.27	94.46	39.82	88.98
**RB_2**	49.64	48.78	46.11	94.53	43.51	89.19
**RB_3**	46.61	45.81	43.18	94.27	40.76	88.99

OBT, Telencephalon (including olfactory bulbs). Hy, Hypothalamus. OT, Optic Tectum. Ce, Cerebellum. Mo, Medulla Oblongata. RB, Remaining Brain.

**Figure 4:**
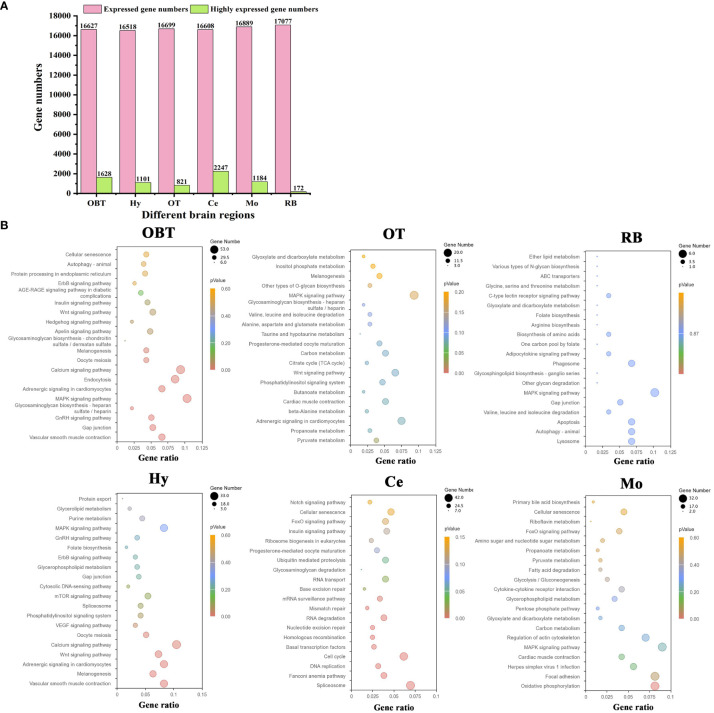
The highly expressed genes for six brain regions and related functions. **(A)** Numbers of expressed and highly expressed genes screened in six brain regions (FPKM value ≥ 1). **(B)** Top 20 enriched KEGG pathways based on highly expressed genes in six brain regions. The gene ratio represented the ratio between the number of genes in a particular pathway and the number of all genes in a certain enrichment term. The sizes of the dots on these plots denoted the number of genes, while colors correspond to the *p* value range. OBT, Telencephalon (including olfactory bulbs). Hy, Hypothalamus. OT, Optic Tectum. Ce, Cerebellum. Mo, Medulla Oblongata. RB, Remaining Brain.

A total of 31419 genes were identified and annotated from all brain transcriptomes, accounting for 83.51% of the whole genome. Among them, 19676 genes (52.30%) were detected with an FPKM value of 1 or higher. The top highly expressed genes in the brain were screened, and the top five genes were cytochrome c oxidase subunit I (*cox1*), cytochrome c oxidase subunit II (*cox2*), cytochrome c oxidase subunit III (*cox3*), cytochrome b (*cytb*), and ATP synthase subunit 6 (*atp6*). Most of these genes were related to energy metabolism, particularly oxidative phosphorylation. Additionally, genes related to neural excitation and transmission were also identified, such as synaptosome-associated protein, excitatory amino acid transporter, GABA receptor-associated protein, and glutamate receptor. Furthermore, a functional enrichment analysis using KEGG was conducted on the top 30 highly expressed genes. The results showed significant enrichment in the process of oxidative phosphorylation (**Figure 3B**). When analyzing the brain regions specifically, 16627, 16518, 16699, 16608, 16889, and 17077 genes were detected with an FPKM value of 1 or higher in the brain regions of OBT, Hy, OT, Ce, Mo and RB, respectively (**Figure 4A**). Notably, the top highly expressed genes in each brain region were identical to those found in the whole brain, including cytochrome c oxidase subunit I, II and III.

### Highly expressed genes in six specific brain regions and their functional predictions

By comparing gene expression levels among the six brain regions, a total of 7153 region-highly expressed genes (FPKM ≥ 1, and higher than the expression in all other brain regions) were screened. Specifically, 1628, 1101, 821, 2247, 1184 and 172 region-highly expressed genes were identified in the brain regions of OBT, Hy, OT, Ce, Mo and RB, respectively (**Figure 4A**). Based on these genes, the functions of each brain region were analyzed using KEGG pathway enrichment. The top 20 KEGG pathways in each brain region were shown in **Figure 4B**. In the OBT brain region, genes such as growth associated protein (*gap43*), somatostatin (*sst1*), and gonadotropin-releasing hormone (*GnRH*) were highly expressed. Eight KEGG pathways associated with growth and reproduction were enriched, including the GnRH signaling pathway, MAPK signaling pathway, Calcium signaling pathway, Oocyte meiosis, and Wnt signaling pathway. In the Hy brain region, genes such as calcium/calmodulin-dependent protein kinase (*camk*), frizzled 5 (*fzd5*), and insulin-like growth factor-binding protein 5 (*igfbp5*) were highly expressed. Nine KEGG pathways related to growth and reproduction were enriched, including the Wnt signaling pathway, Calcium signaling pathway, Oocyte meiosis, VEGF signaling pathway, GnRH signaling pathway, and MAPK signaling pathway, indicating shared functions between these two brain regions. In the OT brain region, genes such as glutamine synthetase (*gs*), glutamate decarboxylase (*gad*), and 4-aminobutyrate aminotransferase (*gabat*) were highly expressed. Most of the KEGG pathways (thirteen) were related to energy metabolism, including seven carbohydrate metabolism pathways (Pyruvate metabolism, Carbon metabolism, Glycosaminoglycan biosynthesis, Other type of O-glycan biosynthesis, Inositol phosphate metabolism, Glyoxylate and dicarboxylate metabolism, and Citrate cycle) and five amino acid metabolism pathways (Beta-alanine metabolism, Butanoate metabolism, Taurine and hypotaurine metabolism, Alanine, aspartate and glutamate metabolism, Valine, leucine and isoleucine degradation), as well as one lipid metabolism pathway (Propanoate metabolism). In the Mo brain region, genes such as myelin basic protein (*mbp*), syntaxin binding protein (*stxbp*) and class I histocompatibility antigen (*hla*) were highly expressed. Twelve energy metabolism-related KEGG pathways were also enriched, including seven carbohydrate metabolism pathways (Oxidative phosphorylation, Carbon metabolism, Pentose phosphate pathway, Glycolysis/Gluconeogenesis, Pyruvate metabolism, Amino sugar and nucleotide sugar metabolism, and Glyoxylate and dicarboxylate metabolism), four lipid metabolism pathways (Glycerophospholipid metabolism, Fatty acid degradation, Primary bile acid biosynthesis, and Propanoate metabolism), and one vitamin metabolism pathway (Riboflavin metabolism). Additionally, two KEGG pathways associated with immune process (Herpes simplex virus 1 infection and Cytokine-cytokine receptor interaction) were annotated. In the RB brain region, genes such as isotocin neurophysin (*itnp*), vasotocin neurophysin (*vtnp*), and thyrotropin-releasing hormone (*trh*) were highly expressed. There were also twelve energy metabolism-related KEGG pathways enriched, including four amino acid metabolism pathways (Valine, leucine and isoleucine degradation, Biosynthesis of amino acids, Arginine biosynthesis, and Glycine, serine and threonine metabolism), three lipid metabolism pathways (Glycosphingolipid biosynthesis, Adipocytokine signaling pathway, and Ether lipid metabolism), three carbohydrate metabolism pathways (Other glycan degradation, Various types of N-glycan biosynthesis, and Glyoxylate and dicarboxylate metabolism), and two vitamin metabolism pathways (One carbon pool by folate and Folate biosynthesis). In the Ce brain region, genes such as neurogenic differentiation factor (*neurod*), cerebellin-1 (*cbln*), and serine/arginine-rich splicing factor 5 (*srsf5*) were highly expressed. Unlike the other five brain regions, the KEGG pathways in the Ce region were primarily associated with the synthesis and repair of DNA and RNA processes, including Spliceosome, Fanconi anemia pathway, DNA replication, Cell cycle, Basal transcription factors, Homologous recombination, Nucleotide excision repair, RNA degradation, Mismatch repair, mRNA surveillance pathway, Base excision repair, and RNA transport pathways. Overall, the “MAPK signaling pathway” was enriched in all brain regions except Ce. The pathways of “Gap junction” (in OBT, Hy, and RB), “Adrenergic signaling in cardiomyocytes”, “Melanogenesis”, “Wnt signaling pathway” (in OBT, Hy, and OT), “Cellular senescence” (in OBT, Ce, and Mo), and “Glyoxylate and dicarboxylate metabolism” (in OT, RB, and Mo) were shared among three brain regions.

### Specifically expressed genes and candidate markers for the six brain regions

**Figure 5:**
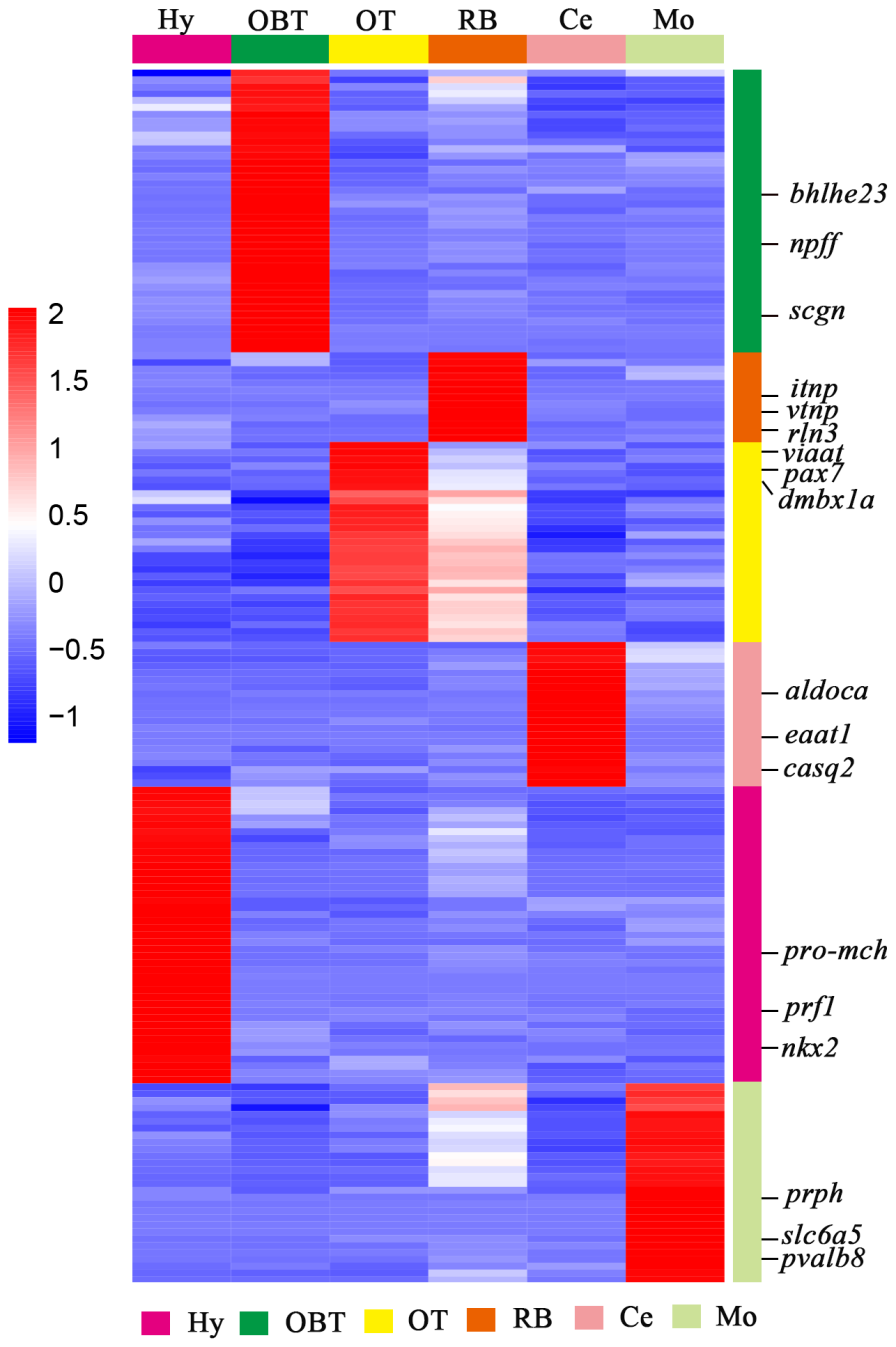
The heatmap analysis of expression level of specifically expressed genes for six brain regions. Each column represented one brain region and each row represents one gene (as colored legend for different brain regions). The color of each gene was shown based on its expression level (RPKM value). Three candidate marker genes screened from each brain region were labeled on the right side.

To explore the specifically expressed genes of different brain regions, the genes expressed at significantly higher level (10 folds) in the target region compared to other regions were further screened and analyzed. A total of 41 specific expressed genes were identified in the OBT region with FPKM values exceeding 10 folds compared to those in other brain regions. Similarly, 43, 29, 21, 29 and 13 specific expressed genes were screened from the Hy, OT, Ce, Mo, and RB regions, respectively (**Figure 5**). Among these specific expressed genes, candidate marker genes were further identified based on their enrichment in respective brain regions compared to other regions, and their FPKM values were shown in **Table 3**. These included class E basic helix-loop-helix protein 23 (*bhlhe23*), neuropeptide FF (*npff*), and secretagogin (*scgn*) as candidate marker genes for the OBT region; pro-melanin-concentrating hormone (*pro-mch*), perforin-1 (*prf1*), and NK2 homeobox (*nkx2*) as candidate marker genes for the Hy region; vesicular inhibitory amino acid transporter (*viaat*), paired box protein Pax 7 (*pax7*), and diencephalon/mesencephalon homeobox protein 1A (*dmbx1a*) as candidate marker genes for the OT region; fructose-bisphosphate aldolase C (*aldoca*), excitatory amino acid transporter 1 (*eaat1*), and calsequestrin-2 (*casq2*) as candidate marker genes for the Ce region; peripherin (*prph*), solute carrier family 6 member 5 (*slc6a5*), and parvalbumin 8 (*pvalb8*) as candidate marker genes for the Mo region; and isotocin neurophysin (*itnp*), vasotocin neurophysin (*vtnp*), and relaxin 3 (*rln3*) as candidate marker genes for the RB region.

**Table 3:** The expression levels (FPKM values) of candidate specific markers in different brain regions.

Gene name	Description	FPKM value					
		OBT	Hy	OT	Ce	Mo	RB
***bhlhe23***	Class E basic helix-loop-helix protein 23	140.46	2.33	1.62	2.20	2.61	2.45
***npff***	Neuropeptide FF	47.45	0.02	0	0	0.08	0.11
***scgn***	Secretagogin	37.76	0.39	0.08	0.12	0.13	0.36
***pro-mch***	Pro-melanin-concentrating hormone	0	212.73	0	0.06	0.03	2.30
***prf1***	Perforin-1	1.05	108.76	0.53	0	0.24	2.58
***nkx2***	NK2 homeobox	0.04	27.22	0	0	0	0.08
***viaat***	Vesicular inhibitory amino acid transporter	0.01	5.09	77.56	0.11	0.09	34.04
***pax7***	Paired box protein Pax 7	0.22	0.06	38.73	0.20	0.72	8.30
***dmbx1a***	Diencephalon/mesencephalon homeobox protein 1A	0	0.05	20.98	0.32	1.54	4.71
***aldoca***	Fructose- bisphosphate aldolase C	0.03	0.02	0.10	219.14	4.05	0.46
***eaat1***	Excitatory amino acid transporter 1	0.52	0.57	0.33	99.05	1.80	1.04
***casq2***	Calsequestrin-2	0.08	0.11	0.28	87.54	3.56	0.80
***prph***	Peripherin	0.86	2.88	0.94	0.13	178.68	32.82
***slc6a5***	Solute carrier family 6 member 5	0.14	0.38	0.51	0.11	22.71	2.57
***pvalb8***	Parvalbumin 8	0	0	0.02	0	11.27	0.14
***itnp***	Isotocin neurophysin	0	1.36	0	0	0.03	3442.64
***vtnp***	Vasotocin neurophysin	0	0.83	0.02	0	0.42	815.45
***rln3***	Relaxin 3	0.85	0.36	0	0.06	0.15	18.75

OBT, Telencephalon (including olfactory bulbs). Hy, Hypothalamus. OT, Optic Tectum. Ce, Cerebellum. Mo, Medulla Oblongata. RB, Remaining Brain.

### Predicted distribution of neurotransmitter-type neurons in the six brain regions

**Figure 6:**
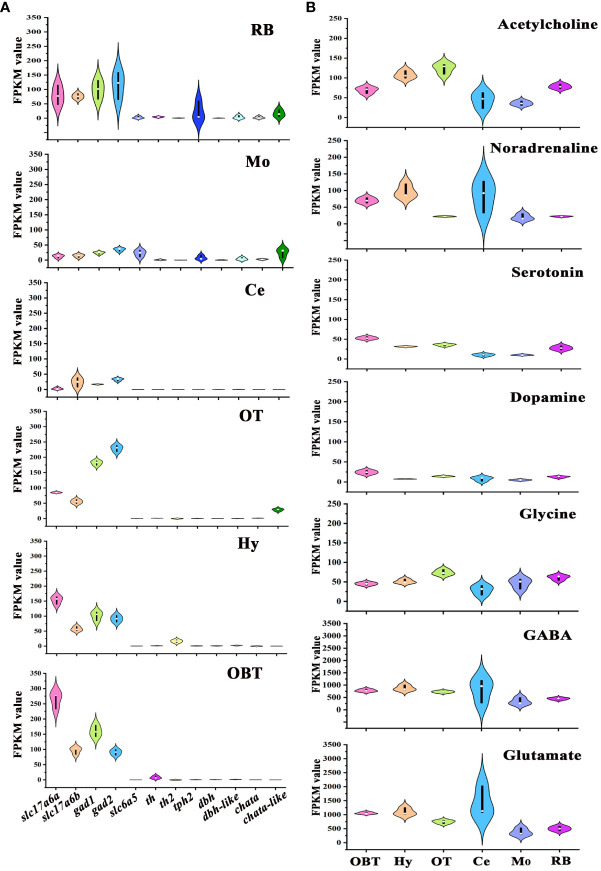
Expression levels of marker genes of seven types of neurons in six brain regions. **(A)** The expression levels of neurotransmitter-type neurons’ markers in six brain regions. **(B)** Total expression levels of seven common neurotransmitters’ receptors in six brain regions. OBT, Telencephalon (including olfactory bulbs). Hy, Hypothalamus. OT, Optic Tectum. Ce, Cerebellum. Mo, Medulla Oblongata. RB, Remaining Brain. *Slc17a6a* and *slc17a6b* are the markers for glutamatergic neurons. *Gad1* and *gad2* are the markers for GABAergic neurons. *Slc6a5* is the marker for glycinergic neurons. *Th* and *th2* are the markers for dopaminergic neurons. *Tph2* is the marker for serotonergic neurons. *Dbh* and *dbh-like* are the markers for noradrenergic neurons. *Chata* and *chata-like* are the markers for cholinergic neurons. The values of the ordinates of the graphs represent RPKM values, and the colors used in the graph are solely for aesthetic purpose, without any specific meaning.

To determine the distribution of neurons synthesizing different neurotransmitters across various brain regions, the marker genes of seven neurotransmitter-type neurons, specifically glutamatergic neuron (*slc17a6*), GABAergic neuron (*gad*), glycinergic neuron (*slc6a5*), dopaminergic neuron (*th*), serotonergic neuron (*tph2*), noradrenergic neuron (*dbh*), and cholinergic neuron (*chata*) were screened from the transcriptomes of six brain regions, referencing data from the model zebrafish (20). In all brain regions, the expression levels of glutamatergic (*slc17a6a*, *slc17a6b*) and GABAergic (*gad1*, *gad2*) marker genes were significantly higher compared to other neurotransmitter types. And the glutamatergic marker genes were particularly abundant in the OBT region, while the GABAergic marker genes were predominantly expressed in the OT region (**Figure 6A**). The expression of glycinergic marker gene (*slc6a5*) was largely detected in the Mo region. The marker genes for dopaminergic neurons (*th and th2*) were mostly expressed in the brain regions of OBT, Hy and RB. The marker gene for serotonergic neurons (*tph2*) was mainly expressed in the RB and Mo. The expression of noradrenergic marker genes (*dbh and dbh-like*) was primarily detected in the RB, Mo and Hy. The expression of cholinergic marker genes (*chata and chata-like*) was primarily detected in the OT, RB and Mo (**Figure 6A**).

The distributions of receptors for these neurotransmitters in different brain regions were also analyzed (**Figure 6B**). The receptor genes of seven neurotransmitters- Glutamate, GABA, Glycine, Dopamine, Serotonin, Noradrenaline, and Acetylcholine- were screened, and the total expressions of receptors for one neurotransmitter in six brain regions of largemouth bass were further analyzed. The expression levels of Glutamate and GABA receptors were higher than those of other neurotransmitters receptors in all six brain regions. The highest expression of noradrenaline receptors was detected in the Hy region (FPKM=105.53). On the other hand, the highest expressions of receptors for glycine and acetylcholine were both detected in the OT region (FPKM values were 74.06 and 124.08, respectively), while the highest expressions of receptors for dopamine and serotonin were both detected in OBT region (FPKM values were 24.38 and 66.92, respectively) (**Figure 6B**).

### Predicted distribution of non-neuronal cells in the six brain regions

**Figure 7:**
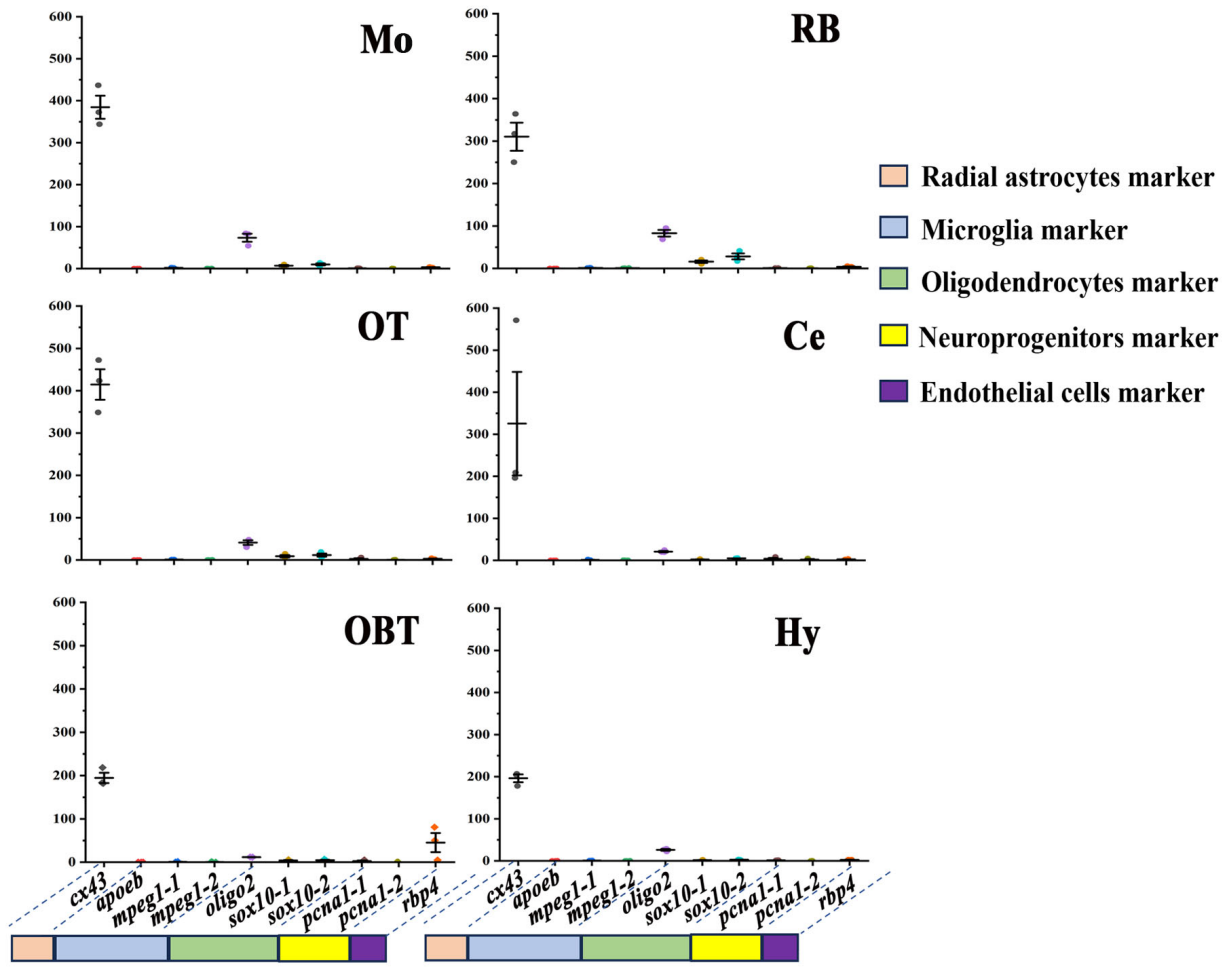
Expression levels of marker genes of non-neuronal cells in six brain regions. OBT, Telencephalon (including olfactory bulbs). Hy, Hypothalamus. OT, Optic Tectum. Ce, Cerebellum. Mo, Medulla Oblongata. RB, Remaining Brain. *Cx43* is the marker for radial astrocytes. *Apoeb*, *mpeg1-1*, and *mpeg1-2* are the markers for microglia. *Oligo2*, *sox10-1*, and *sox10-2* are the markers for oligodendrocytes. *Pcna1-1* and *pcna1-2* are the markers for neuroprogenitors. *Rbp4* is the marker for endothelial cells.

The distribution of non-neuronal cells in different brain regions was also predicted by the expression level of related marker genes. According to putative marker genes in zebrafish (20), the marker genes of five non-neuronal cells, including oligodendrocytes (*olig2*, *sox10*), microglia (*apoeb*, *mpeg1*), endothelial cells (*rbp4*), radial astrocytes (*cx43*), and neuroprogenitors (*pcna*), were identified in the transcriptomes of largemouth bass brain. Among them, the marker genes for *sox10*, *mpeg1*, and *pcna* revealed that all three possess two homologous genes annotated with identical names and functions in the transcriptional data derived from the largemouth bass. To facilitate their distinction, two version (-1 and -2) were employed to classify them. In all six brain regions, the marker gene for radial astrocyte (*cx43*) was much higher expressed (especially in Mo and OT) than other marker genes (**Figure 7**). The expressions of marker genes for oligodendrocyte (*olig2*, *sox10-1*, and *sox10-2*) were secondarily higher in the six brain regions (especially in Mo and RB) than those of the rest of the cell type markers. However, the expressions of the marker genes for microglia (*apoeb*, *mpeg1-1, mpeg1-2*) were nearly undetectable in all six brain regions. The marker genes for neuroprogenitor (*pcna-1, pcna-2*) and endothelial (*rbp4*) were primarily expressed in Ce and OBT, respectively (**Figure 7**).

### Relative expression of marker genes in the six brain regions

**Figure 8:**
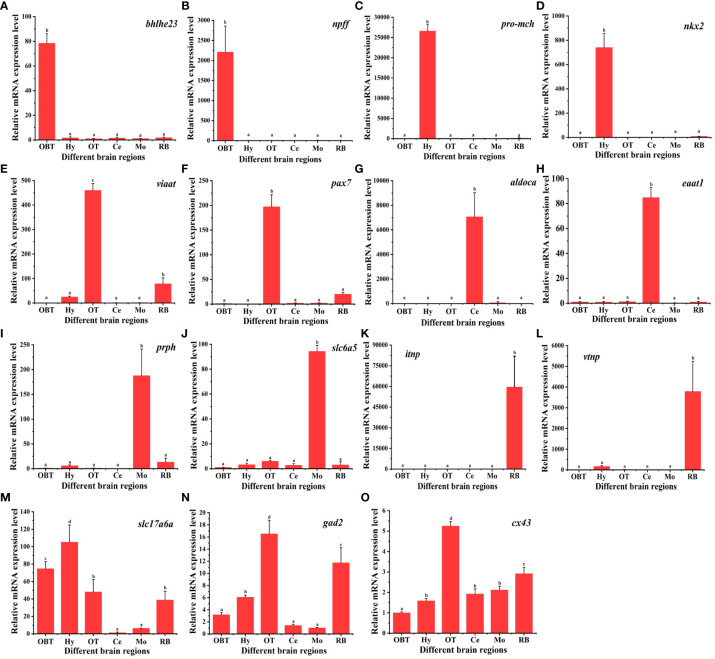
The relative expression levels of marker genes in six brain regions of largemouth bass. Fifteen marker genes were selected for qPCR validation of RNA-seq data. *Bhlhe 23* and *npff* are the candidate marker genes for the OBT region. *Pro-mch* and *nkx2* are the candidate marker genes for the Hy region. *Viaat* and *pax7* are the candidate marker genes for the OT region. *Aldoca* and *eaat1* are the candidate marker genes for the Ce region. *Prph* and *slc6a5* are the candidate marker genes for the Mo region. *Itnp* and *vtnp* are the candidate marker genes for the Hy region. *Slc17a6a* is the marker for glutamatergic neurons. *Gad2* is the marker for GABAergic neurons. *Cx43* is the marker for radial astrocytes. OBT, Telencephalon (including olfactory bulbs). Hy, Hypothalamus. OT, Optic Tectum. Ce, Cerebellum. Mo, Medulla Oblongata. RB, Remaining Brain.

A total of 15 marker genes were selected for qPCR validation of RNA-seq data. These comprised 12 candidate marker genes for the six brain regions, two marker genes for glutamatergic and GABAergic neuron, and one for radial astrocytes. As shown in **Figure 8**, the candidate marker genes for the OBT brain region, *bhlhe*23 and *npff*, exhibited extremely significant expression specifically in the OBT region. Similarly, *pro-mch* and *nkx2*, candidate markers for the Hy brain region, were significantly expressed in the Hy region. The *viaat* and pax7, candidate markers for the OT brain region, displayed extremely significant expression patterns in the OT region (*p* < 0.01). The *aldoca* and *eaat1*, candidate markers for the Ce brain region, were also extremely significantly expressed in the Ce region. The *prph* and *slc6a5*, candidate markers for the Mo brain region, were significantly expressed in the Mo region. The *itnp* and *vtnp*, candidate markers for the RB brain region, exhibited extremely significant expression in the RB region (*p* < 0.01). Furthermore, the glutamatergic marker gene *slc17a6b* was significantly enriched in the Hy and OBT regions, with lower expression in the Ce and Mo regions (*p* < 0.01). Similarly, the GABAergic marker gene *gad2* was significantly enriched in the OT and RB regions, while exhibiting less enrichment in the Ce and Mo regions (*p* < 0.01). Additionally, the radial astrocyte marker gene *cx43* was significantly overexpressed in the OT region (*p* < 0.05). Taken together, these results demonstrated that the expression patterns determined by qPCR closely align with those obtained through RNA-Seq, thereby validating the reliability and accuracy of the RNA-seq data.

## Discussion

The brain is the core of the central nervous system in vertebrates. It is a complex organ composed of different subregions, each of which performs specific functions in regulating growth, reproduction, metabolism, movement, cognition and sensation (10). However, compared with the extensive knowledge of brain structure and function in mammals, the understanding of those in teleost, which is the largest group of vertebrates, is still significantly lacking. Although the brain structure and function of various fish species, including zebrafish (*Danio rerio*), grass carp (*Ctenopharyngodon idellus*), sea bass (*Dicentrarchus labrax*), mudskipper (*Boleophthalmus pectinirostris*), tilapia (*Oreochromis mossambicus*), and african turquoise killifish (*Nothobranchius furzeri*), among others, have been extensively explored, further attempts are still necessary to uncover the commonalities and variations among different aquaculture species (7–12, 20, 31). This will not only pave the way for a deeper understanding of the regulatory role of the brain in fish, but also provide a reference for research in comparative neurobiology during evolution. Largemouth bass is one of the most important aquaculture species in China, and research on its brain morphology and related functions is crucial for better understanding its basic physiological characteristics. Some studies have explored the functions of hypothalamus under environmental stress (32, 33), but little is known about the functions of other brain regions in the largemouth bass. Therefore, the structure and function of its brain were further explored in our present study using histological observation and transcriptome analysis.

A global view of the anatomical structure of the brain in largemouth bass was captured for the first time under an anatomical lens. As expected, four brain parts-the telencephalon, diencephalon, mesencephalon, and rhombencephalon-were successfully identified, exhibiting similar anatomical features to those observed in other teleosts. Olfactory bulbs were found to locate at the forefront of the brain in largemouth bass and directly adhered to the telencephalic hemispheres, which were quite different from those in cyprinid fish. In the latter, such as goldfish (*Carassius auratus*) and grass crap (*Ctenopharyngodon idellus*), the olfactory bulbs connected with the telencephalic hemispheres via long olfactory tracts (10, 34). The lack of long olfactory tracts in largemouth bass has also been reported in most perciform species, including the sea bass (*Dicentrarchus labrax*), mudskipper (*Boleophthalmus pectinirostris*), and Cichlid (*Oreochromis mossambicus*) (7–9). As expected, the optic tectum, belonging to the mesencephalon, was located at the caudal of the telencephalic hemispheres. Interestingly, the size of the optic tectum in largemouth bass was proportionally larger than other brain parts, suggesting a highly developed visual system in largemouth bass. Almost all of the rhombencephalon (including cerebellum and medulla oblongata) was located in the caudal of the optic tectum, except for the valvula cerebelli of the cerebellum, which was wrapped in the optic tectum. This is consistent with the external morphology of the brain in grass carp (*Ctenopharyngodon idellus*), zebrafish (*Danio rerio*) and mudskipper (*Boleophthalmus pectinirostris*) (9–11). However, unlike the position of the cerebellum in sea bass *Dicentrarchus labrax* (15), part of corpus cerebelli of the cerebellum was also located on the dorsal of the optic tectum. In the present study, the saccus vasculosus was also identified in largemouth bass and located in the ventral of hypothalamus. Based on the reported function of saccus vasculosus, it might participate in regulating adaption to changes in circadian or seasonal rhythms in largemouth bass (35). Collectively, the anatomical structure of the brain in largemouth bass is relatively conserved compared to those of other teleosts, yet it also possesses its own characteristics, such as the absence of olfactory tracts, the possession of a large optic tectum, and the presence of a saccus vasculosus.

In addition to the external morphology analysis, several nuclei were identified in the internal structure of largemouth bass brain. Based on the nomenclature adopted in previous study (8, 9, 15, 26), a total of 74 nuclei were identified in the four brain parts after HE staining. In the telencephalon, there were 16 nuclei, including the Dm, Dl, VV, and Dc nucleus. The nucleus of Dm, Dl, and VV in fish have been reported as functional homologues to the mammalian amygdala, hippocampus, and lateral septum regions, which were involved in regulating anxiety or depression (16). Besides, the nucleus of Dc in gymnotiform fish has been reported to participate in learning and memory (36). These identified nuclei in the telencephalon area of largemouth bass provide a foundation for future studies on the physiological functions related to emotion, learning, and memory. In the mesencephalon, the optic tectum is the main area responsible for and processing of visual signal (37). Besides, numerous visual-related nuclei were also present in the diencephalon of largemouth bass, such as the magnocellular preoptic nucleus in the preoptic area, nucleus glomerulosus in the posterior tuberculum area, parvocellular superficial pretectal nucleus, and central pretectal nucleus in the pretectum area. These further indicated that largemouth bass possesses a well-developed visual system. In our present study, the pineal gland, an important light sensor, was also identified in the dorsal of the diencephalon, and it connected to the habenula through the pineal stalk and habenular commissure. Recent reports have been showed that the habenula played a crucial role as a center for emotion-associated adaptation behaviors (38). The interconnection of the pineal gland and habenula suggests a possible way of using light to regulate behaviors in fish. In the rhombencephalon, the connection between the valvula cerebelli (VCe) in the cerebellum and the torus longitudinalis (TLo) in the mesencephalon is controversial in teleost. In sea bass (*Dicentrarchus labrax*), the cells in the granular layer of VCe seemed to be continuous with those of the lateroventral of TLo. In sea bream (*Sparus aurata*), a tract has been detected between the VCe and TLo, and the existence of this tract has also been confirmed in zebrafish (15, 39). By contrast, VCe and TLo in largemouth bass were neither continuous with each other nor did they have a tract between them. Moreover, the cells in the granular layer of the VCe were smaller and darker than the outermost layer of the TLo. Those results suggest that VCe is not connected to TLo in largemouth bass. These subtle nuclei identified suggest an elaborate development and complex function of the brain in largemouth bass.

Based on structural composition, six brain regions of largemouth bass were separated, and their potential functions were explored through gene expression analysis. Some studies have predicted the functions of different brain regions in fish based on the expression features of specific genes (40, 41). However, the expression characteristics of few genes cannot fully reflect the real function of various brain regions. To obtain an overall gene expression pattern, a transcriptome sequencing method was employed to detect gene expression levels in six brain regions (OBT, Hy, OT, Ce, Mo, and RB) of largemouth bass. In our current study, it was found that oxidative phosphorylation- related genes were the most highly expressed in the whole brain of largemouth bass, indicating that the energy consumption was highly demanded in brain, which is consistent with the repots stating that the brain is one of the most energetically expensive organs in the vertebrate body (42–44). The top 20 KEGG pathways in each brain region were also analyzed. Interestingly, ten pathways were shared between the brain region of OBT and Hy, most of which were related to growth and reproduction. In grass carp (*Ctenopharyngodon idellus*), the telencephalon and hypothalamus have been reported to be highly correlated and play center roles in feeding and reproduction (10). It was generally accepted that the telencephalon and hypothalamus in teleost developed from a common anterior neural plate (45). In addition, the inferior lobe of the hypothalamus can receive nerve afferents from the Dm area of the telencephalon in many teleost species, such as in *Cyprinus*, *Danio*, and *Oncorhynchus* (46). These results suggest that the OBT and Hy regions play similar roles in regulating growth and reproduction. On the other hand, the KEGG pathways enriched in OT, RB, and Mo regions were mostly related to metabolism processes, including carbohydrate, amino acid, lipid, and vitamin metabolism. Consistently, in the Zebra finch (*Taeniopygia guttata*), metabolism pathways for glucose, pyruvate, glyoxylate, dicarboxylate, alanine, and aspartate were also enriched in the optic tectum (47). Furthermore, unlike the other five brain regions, more amino acid metabolism pathways were identified in the OT region. Amino acid metabolism has been reported to play crucial roles in retinal function (48). It is speculated metabolism processes in the OT region play a crucial role in regulating visual function. In the Mo region, there were two immune-related pathways (Herpes simplex virus 1 infection and Cytokine-cytokine receptor interaction) that were enriched, indicating its immune-modulating function. Unlike mammals, fish can synthesize folate themselves, and two folate-related pathways (One carbon pool by folate and Folate biosynthesis) were enriched in the RB regions, suggesting RB region is the main brain area for folate synthesis in largemouth bass (49). Although no common pathway was found in all six brain regions, the MAPK signaling pathway was shared in five brain regions (OBT, Hy, OT, Mo, and RB). The widespread distribution of the MAPK pathway implies its fundamental roles in the brain of largemouth bass, similar to that in mammals (50).

To further elucidate the distinct roles of various brain regions, a screening process was conducted to identify specifically expressed genes and putative molecular marker genes in each brain region. In the OBT region, genes related to growth and reproduction, such as insulin-like growth factor binding protein 5 (*igfbp5a*) and gonadotropin releasing hormone III (*gnrh3*), were abundantly expressed. Neuropeptide FF (*npff*), identified as a potential marker for OBT, has also been implicated in the regulation of reproduction in fish (51). Additionally, neurotransmitter receptors associated with emotion, learning and memory, such as serotonin receptor and adrenergic receptor, were highly expressed in the OBT (52–54). Collectively, these finding suggest that the OBT brain region in largemouth bass primarily functions in regulating growth, reproduction, emotion, learning, and memory.

In the Hy region, the feeding-related neuropeptides pro-melanin-concentrating hormone (*pro-MCH*) and agouti-related protein (*AgRP*) were significantly enriched. This finding is consistent with the hypothalamus’s role in controlling feeding and satiety in fish (55, 56). Furthermore, the highly expressed stress-related genes in the Hy, including ACTH precursor peptide pro-opiomelanocortin (*pomc*), synthetase of catecholamine tyrosine hydroxylase 2 (*th2*), and transcription factor of *pomc* NK2 homeobox (*nkx2*), suggest that the Hy plays a crucial role in regulating the stress response (57, 58). Together with the KEGG pathways enriched in the Hy as mentioned above, our results indicate that the Hy brain region in largemouth bass may not only function in growth and reproduction, but also serves as the center for regulating feeding and stress response.

In the OT region, the box protein Pax 7 (*pax7*) and diencephalon/mesencephalon homeobox protein 1A (*dmbx1a*) were selected as candidate marker genes due to their abundant expression in OT. They are consistent with reported results in zebrafish and grass carp (10, 59). Both of these genes are closely related to the development of the visual system. Additionally, several vision-related genes were also abundant in OT, including brain-specific homeobox/POU domain protein 3b (*brn3b*), which is crucial for retinal ganglion cell differentiation, cellular retinaldehyde-binding protein (*cralbp*), which is a marker for retinal müller cell, Opsin-3 (*opn3*), which is a light sensor, and retinal dehydrogenase 1 (*raldh1*), which is retinoic acid synthesizing enzyme and crucial for the developing of the optic tectum (60–64). Collectively, these results suggest that the OT brain region in largemouth bass plays a critical role in regulating the visual system.

In the Ce region, fructose-bisphosphate aldolase C (*aldoca*), excitatory amino acid transporter 1 (*eaat1*), and calsequestrin-2 (*casq2*) were specifically expressed. As reported in mammals, both *aldoca* and *casq2* were highly expressed in purkinje cells of the cerebellum, while *eaat1* was expressed in astrocytes of the cerebellum (65–67). The genes retinoschisin (*rs*) and betaine-homocysteine S-methyltransferase (*bhmt*) were also enriched in the Ce region of largemouth bass, playing roles in the visual and hearing system, respectively (68, 69). These results suggest that the Ce brain region in largemouth bass is primarily involved in sensory integration and receives inputs primarily from the visual and auditory systems.

In the Mo region, peripherin (*prph*), parvalbumin 8 (*pvalb8*), and solute carrier family 6 member 5 (*slc6a5*) were identified as candidate markers, all of which were related to sensor, respiratory, and somatic motor functions. *Prph* was reported to be expressed in the sensory and motor neurons of mice (70). *Pvalb8* was largely distributed in the ventrolateral medulla of rat, particularly in respiratory neurons of the ventrolateral medulla, and regulated respiratory processes (71). *Slc6a5*, as a marker of the Glycinergic system, was also reported to participate in the processing of motor and sensory information that underlies movement, vision and audition in mammals (72). Additionally, paired mesoderm homeobox (*phox2b*) was also enriched in the Mo region and involved in visceral sensory input and motor output (73). Collectively, these findings suggest that the Mo region in largemouth bass is involved in processing sensory information, as well as regulating respiration and somatic motor functions.

In the RB brain region, genes of isotocin neurophysin (*itnp*) and vasotocin neurophysin (*vtnp*) were highly enriched, and they have well-established effects on osmoregulation and stress response in fish (74). In zebrafish, it was reported that *itnp* and *vtnp* are distributed in preoptic area (75, 76). And in our study, the preoptic area was included in the RB region, indicating *itnp* and *vtnp* might be highly expressed in the preoptic area of largemouth bass. Relaxin 3 (3a and 3b) were also significantly enriched in the RB brain region, and they were also abundant in the middle-posterior regions of the eel brain (77). The function of relaxin 3 in mammals suggested it plays a role in sleep/wake states, as well as stress response (78). Furthermore, reproduction-related genes such as progonadoliberin-1 (*gnrh1*) and gonadotropin-releasing hormone II (*gnrh2*) were also highly expressed in the RB region. All these results suggest that the RB brain region in largemouth bass might participate in regulating osmoregulation, stress response, sleep/wake states, and reproduction.

The heterogeneous distribution of neurons in different brain regions is already well known in mammalian brains, and neurotransmitters are the key functional performers for diverse neurons types (79). However, the distribution characteristics of different neurotransmitters and their related neuron types are largely unknown in the brain of aquaculture fish species. In the present study, the expression patterns of marker genes for seven neurotransmitter-type neurons (glutamatergic, GABAergic, glycinergic, dopaminergic, serotoninergic, noradrenergic, and cholinergic) were analyzed. The highly expressed glutamatergic and GABAergic marker genes in all brain regions indicated that they are necessary for normal brain functioning. These results were consistent with reports in mammals that glutamate and GABA were the primary excitatory and inhibitory neurotransmitters in the central nervous system (80, 81). In addition, some markers of neurotransmitter-type neurons were screened as specifically expressed genes in specific brain region of largemouth bass, such as dopamine synthetase *th2* highly expressed in the Hy region, glycine transporter *slc6a5* widely enriched in the Mo region, suggesting the heterogeneous distribution characteristics of neurons also exist in the brain of aquaculture fish.

Neurotransmitters function by binding to their related receptors. Uncovering the regional expression of various neurotransmitter receptors is also crucial for deeply understanding brain function (82). In our present study, the total expressions of each neurotransmitter receptors in different brain regions of largemouth bass were also explored. In agreement with the widespread expressions of Glutamatergic and GABAergic marker genes in six brain regions, their receptors were also highly expressed in all brain regions, suggesting their importance. In contrast, the expressions of glycine receptors were mainly distributed in the OT region, contracting with its synthesizing region (Mo) of glycine, indicating a paracrine function for glycine. Similarly, the specific distributions of serotonin receptors in the OBT region indicated a paracrine manner of serotonin derived from the Mo and RB regions. These results were quite different from the findings in Atlantic salmon (*Salmo salar*), where both serotonin and its receptors displayed higher expression levels in the Dm region of the telencephalon and functioned in an autocrine manner (6). As for dopamine, noradrenaline, and acetylcholine, they, as well as their receptors, were mainly expressed in brain regions of OBT, Hy, and OT, respectively, and similar patterns of autocrine function were revealed.

Non-neuronal cells are also cellular components of the brain and involved in a diverse range of functions, such as neurogenesis, synaptogenesis, synaptic plasticity, metabolic process, and stress (83, 84). It has been found that non-neuronal cells possess an even larger proportion than neurons in some brain regions of adult rats (85). Recently, markers of different types of non-neuronal cells were identified in zebrafish using single-cell transcriptomes sequencing, such as radial astrocytes (*cx43*), oligodendrocytes (*oligo 2*, *sox10-1*, and *sox10-2*), microglia (*apoeb*, *mpeg1-1*, and *mpeg1-2*), neuroprogenitors (*pcna1-1* and *pcna1-2*), and endothelial cells (*rbp4*) (20). Using these markers, the possible distribution of non-neuronal cells in six brain regions of largemouth bass was explored in our present study. The higher expression of *cx43* in all six brain regions indicated the ubiquitous distribution of radial astrocytes in the brain of largemouth bass. While in human, oligodendrocytes have been reported to be more ubiquitous in the adult brain (86). It can be inferred that adult teleost need more radial astrocytes for neurogenesis, whereas this process is nearly disappeared in mammals (87). The marker for radial astrocytes was highest expressed in the OT region, suggesting their distribution was mainly in the OT, which was consistent with findings in other teleosts, such as goldfish (*Carassius auratus*), thicklip grey mullet (*Chelon labrosus*), and tench (*Tinca tinca*) (87). The lower expression of microglia marker genes in all six brain regions suggested a smaller proportion of microglia in largemouth bass brain, similar to mammals (86). Microglia are the tissue-resident macrophages in the central nervous system and are critically involved in immune defense (88). The widespread distribution of microglia marker genes in the Mo region suggested its involvement in immune function, which is consistent with the immune-related KEGG pathways enriched in the Mo region as described above. Besides, marker genes for oligodendrocytes and neuroprogenitors were highest expressed in the RB and Ce region, respectively, indicating their resident in the RB and Ce region, respectively. As all of these results were based on the gene expression of candidate marker genes, more detailed studies are needed to understand the distribution and function of non-neuronal cells in the brain of aquaculture fish.

Fish have evolved unique physiological characteristics to better adapt to their environments, and largemouth bass is no exception. As a freshwater species, largemouth bass can live in coastal water, exhibiting remarkable salinity tolerance (89). Our transcriptome analysis of the largemouth bass brain revealed a high expression of osmoregulation-related genes, particularly isotocin neurophysin and vasotocin neurophysin, in the RB regions. This suggests that these genes might play a crucial role in regulating the salinity tolerance of largemouth bass. Furthermore, given their carnivorous nature and preference for foraging in low light intensity environments (90, 91), it is speculated that a robust visual system might be essential for locating and acquiring mobile prey. Consistent with this hypothesis, our study demonstrates a well-developed visual system in the largemouth bass, evident from the enlarged size of their optic tectum structure and the high neural activity. This is supported by the high expression of marker genes for neuron and non-neuronal cells in the OT region, such as *gad2* and *cx43* (**Figure 8**).

In conclusion, the cytoarchitectonic and gene expression characterization of six brain regions of largemouth bass were uncovered in the present study, filling a gap in the knowledge of brain structure and function of largemouth bass. However, the distribution features of different cell types (neuron, glia, endothelial cells, etc.) in the brain at the cellular level are still unclear, and a more precise understanding of the function of each brain region still needs further exploration. Taken together, these results provide the first global catalogue of morphological structure and function characteristics of distinct brain regions of largemouth bass, which will provide a valuable resource for further study of the regulatory mechanism of specific brain subregions in diverse physical functions in cultured fishes.

## Data availability statement

The data presented in the study are deposited in the Genome Sequence Archive in National Genomics Data Center, China National Center for Bioinformation, accession number is GSA: CRA014769.

## Ethics statement

The animal study was approved by Institutional Animal Care and Use Committee (IACUC) of Zhejiang University (Zhejiang, China). The study was conducted in accordance with the local legislation and institutional requirements.

## Author contributions

ML: Data curation, Writing – original draft, Writing – review & editing. LY: Data curation, Writing – review & editing. LZ: Writing – review & editing, Resources. QZ: Resources, Writing – review & editing. YL: Supervision, Writing – review & editing.

## Supplementary material

Supplementary Figure 1 The dimensions of different brain regions of largemouth bass. **(A)** Length, **(B)** Width, and **(C)** Depth of five brain regions were shown. OB, Olfactory Bulbs. Tel, Telencephalon. OT, Optic Tectum. Mo, Medulla Oblongata. CCe, Corpus Cerebelli.

## Glossary

**Table:**

**Telencephalon region**	
**Dorsal telencephalon subregion**	
Dm2 nucleus	subdivision 2 of the medial part of dorsal telencephalon
Dm3 nucleus	subdivision 3 of the medial part of dorsal telencephalon
Dm4 nucleus	subdivision 4 of the medial part of dorsal telencephalon
Dd nucleus	dorsal part of dorsal telencephalon
Dld nucleus	dorsal division of the lateral part of dorsal telencephalon
Dlp nucleus	posterior division of the lateral part of dorsal telencephalon
Dlv2 nucleus	ventral division of the lateral part of dorsal telencephalon
Dc1 nucleus	central part of dorsal telencephalon
Dc2 nucleus	central part of dorsal telencephalon
Dp nucleus	posterior part of dorsal telencephalon
**Ventral telencephalon subregion**	
Vd nucleus	dorsal nucleus of ventral telencephalon
Vv nucleus	ventral nucleus of ventral telencephalon
Vs nucleus	supracommissural nucleus of ventral telencephalon
Vp nucleus	postcommissural nucleus of ventral telencephalon
Vl nucleus	lateral nucleus of ventral telencephalon
Vc nucleus	central nucleus of ventral telencephalon
**Others**	
Se	sulcus externus
Sy	sulcus ipsilyformis
LFB	lateral forebrain bundles
**Diencephalon region**	
**Preoptic area subregion**	
NPOav nucleus	anteroventral part of the parvocellular preoptic nucleus
NPOpc nucleus	parvocellular part of the parvocellular preoptic nucleus
PMgc nucleus	gigantocellular part of the magnocellular preoptic nucleus
NAPV	anterior periventricular nucleus
**Ventral thalamus subregion**	
VM nucleus	ventromedial thalamic nucleus
VL nucleus	ventrolateral thalamic nucleus
I nucleus	intermediate thalamic nucleus
**Dorsal thalamus subregion**	
A nucleus	anterior thalamic nucleus
**Epithalamus subregion**	
Epi	epiphysis
NHd nucleus	dorsal habenular nucleus
NHv nucleus	ventral habenular nucleus
HaCO	habenular commissure
**Hypothalamus subregion**	
NRLI nucleus	lateral part of the nucleus of the lateral recess
NDLII nucleus	lateral part of the diffuse nucleus of the inferior lobe
NDLIm nucleus	medial part of the diffuse nucleus of the inferior lobe
NCLI nucleus	central nucleus of the inferior lobe
**Posterior tuberculum subregion**	
NPGa nucleus	anterior preglomerular nucleus
NPGm nucleus	medial preglomerular nucleus
NGp nucleus	posterior part of the nucleus glomerulosus
NPGc nucleus	commissural preglomerular nucleus
CM	corpus mamillare
TL nucleus	lateral thalamic nucleus
**Synencephalon subregion**	
nMLF nucleus	nucleus of the medial longitudinal fasciculus
**Pretectum subregion**	
Psp nucleus	parvocellular superficial pretectal nucleus
NPC nucleus	central pretectal nucleus
**Others**	
OT	optic tectum
P	pituitary
SV	saccus vasculosus
HCO	Horizontal commissure
**Mesencephalon region**	
**Dorsal tectum subregion**	
SWGZ	superficial white and gray zone of the optic tectum
CZ	central zone of the optic tectum
DWZ	deep white zone of the optic tectum
PGZ	periventricular gray zone of the optic tectum
TLo	torus longitudinalis
**Ventral tegmentum subregion**	
**Medial zone**	
nlll	nucleus of oculomotor nerve
EW	edinger-Westphal nucleus
NR	nucleus ruber
**Central zone**	
PLm	medial part of perilemniscular nucleus
PLI	lateral part of perilemniscular nucleus
VT	ventral tegmental nucleus
**Lateral zone**	
TSc	central part of semicircular torus
TSl	lateral part of semicircular torus
TSv	ventral part of semicircular torus
**Others**	
Opn	optic nerve
llf	lateral longitudinal fascicle
**Rhombencephalon region**	
**Cerebellum subregion**	
VCe	valvula of the cerebellum
M	molecular layer of the cerebellum
G	granular layer of the cerebellum
Pj	purkinje cells
CCe	corpus of the cerebellum
EG	granular eminence
**Medulla oblongata subregion**	
**Reticular formation**	
SR	superior nucleus of the raphe
RS	superior reticular nucleus
RM	medial reticular nucleus
**Area octavolateralis**	
AON	anterior octaval nucleus
DON	descending octaval nucleus
MON	medial octavolateral nucleus
CC	cerebellar crest
**Somatomotor nuclei**	
**nlV**	trochlear nerve nucleus
**nVlc**	caudal part of abducens nerve nucleus
**Visceromotor nuclei**	
Vm	trigeminal nerve nucleus
VIIm	facial motor nucleus
**Other rhombencephalic nuclei**	
NGS	secondary gustatory nucleus
PE	pre-eminential nucleus
**Others**	
V	trigeminal nerve
