# Supplementary material for: Specific biomarkers and neurons distribution of different brain regions in largemouth bass (Micropterus salmoides)
Source: Front Endocrinol (Lausanne). 2024 Apr 30;15:1385575. doi: 10.3389/fendo.2024.1385575 (PMC11091468; doi:10.3389/fendo.2024.1385575)
Supplement: Supplementary Figure 1 — The dimensions of different brain regions of largemouth bass. (A) Length, (B) Width, and (C) Depth of five brain regions were shown. OB, Olfactory Bulbs. Tel, Telencephalon. OT, Optic Tectum. Mo, Medulla Oblongata. CCe, Corpus Cerebelli. [file Presentation_1.pdf]

Supplementary Material

Figure S1

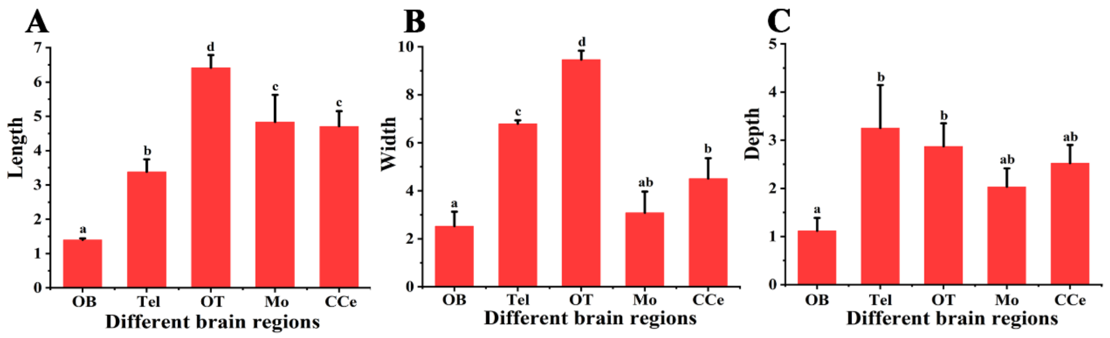

Figure S1. The dimensions of different brain regions of largemouth bass.

(A) Length, (B) Width, and (C) Depth of five brain regions were shown. OB, Olfactory Bulbs. Tel, Telencephalon. OT, Optic Tectum. Mo, Medulla Oblongata. CCe, Corpus Cerebelli.
